# Recommendations for the treatment of epilepsy in adult patients in general practice in Belgium: an update

**DOI:** 10.1007/s13760-012-0070-9

**Published:** 2012-04-28

**Authors:** Paul Boon, Sebastiaan Engelborghs, Henri Hauman, An Jansen, Lieven Lagae, Benjamin Legros, Michel Ossemann, Bernard Sadzot, Katrien Smets, Etienne Urbain, Kenou van Rijckevorsel

**Affiliations:** 1 Department of Neurology, Ghent University Hospital, 185 De Pintelaan, 9000 Ghent, Belgium; 2 Department of Neurology and Memory Clinic, Hospital Network Antwerp (ZNA) Middelheim and Hoge Beuken, Institute Born-Bunge, University of Antwerp, Antwerp, Belgium; 3 Department of Neurology, Sint-Maarten Hospital, Duffel, Belgium; 4 Department of Paediatric Neurology, University Hospital Brussels, Free University Brussels, Brussels, Belgium; 5 Department of Paediatric Neurology, University Hospital, Leuven, Belgium; 6 Department of Neurology, Hôpital Erasme, Université Libre de Bruxelles, Brussels, Belgium; 7 Department of Neurology, University Hospital Mont-Godinne, Université Catholique de Louvain, Yvoir, Belgium; 8 Department of Neurology, University Hospital Centre Sart-Tilman, Liège, Belgium; 9 Department of Neurology, Antwerp University Hospital, University of Antwerp, Antwerp, Belgium; 10 Department of Neurology, Grand Hôpital de Charleroi, Charleroi, Belgium; 11 Department of Paediatric Neurology, Saint Luc University Hospital, Université Catholique de Louvain, Brussels, Belgium

**Keywords:** Epilepsy, Antiepileptic drugs, Seizures, Treatment, Recommendations

## Abstract

In 2008, a group of Belgian epilepsy experts published recommendations for antiepileptic drug (AED) treatment of epilepsies in adults and children. Selection of compounds was based on the registration and reimbursement status in Belgium, the level of evidence for efficacy, common daily practice and the personal views and experiences of the authors. In November 2011 the validity of these recommendations was reviewed by the same group of Belgian epilepsy experts who contributed to the preparation of the original paper. The recommendations made in 2008 for initial monotherapy in paediatric patients were still considered to be valid, except for the first choice treatment for childhood absence epilepsy. This update therefore focuses on the treatment recommendations for initial monotherapy and add-on treatment in adult patients. Several other relevant aspects of treatment with AEDs are addressed, including considerations for optimal combination of AEDs (rational polytherapy), pharmacokinetic properties, pharmacodynamic and pharmacokinetic interaction profile, adverse effects, comorbidity, treatment of elderly patients, AED treatment during pregnancy, and generic substitution of AEDs.

## Introduction

The number of antiepileptic drugs (AEDs) currently registered in Belgium is considerable and still increasing. In the 1970s only 6 AEDs, benzodiazepines excluded, were available: phenobarbital, phenytoin, ethosuximide, primidone, carbamazepine and valproate. Since the 1990s the number of approved AEDs has increased exponentially, though not all of these compounds are marketed (yet).

Although this large choice of AEDs allows tailoring treatment to the individual patient’s needs, it also makes selection of the most suitable compound a complicated task. To provide guidance for the management of epilepsy in general practice in Belgium, a group of experts published recommendations for AED treatment of epilepsies in adults and children in 2008 [1].

The literature and the views of the authors regarding initial monotherapy for seizures in paediatric patients (<16 years) have not changed; the recommendations made in 2008 are therefore still considered valid [1]. The present publication focuses on recommendations for AED treatment of epilepsies in adult patients, providing an update on selection of AEDs for initial monotherapy (for focal seizures, primary generalized seizures and type of seizures not yet established) and for add-on treatment of seizures (focal seizures and primary generalized tonic–clonic seizures).

The choice of an AED primarily depends on the efficacy of the compound for controlling the patient’s seizure type or in a specific epilepsy syndrome. Other patient-specific factors, such as age, presence of concomitant diseases (including hepatic or renal impairment), use of concomitant medication (including use of other AEDs), sex and childbearing potential should also be taken into consideration. Factors such as pharmacokinetics, tolerability and safety of the AED, pharmacokinetic and pharmacodynamic interaction potential, ease of use and dosing frequency, availability of pharmaceutical formulations, and possibility to rapidly obtain seizure control will also play a role in AED selection.

In addition to providing recommendations for treatment, the present paper will also address some of these patient-related and AED-related factors relevant for the selection of the optimal compound.

## Methodology

In 2008, recommendations for the AED treatment of epilepsies in general practice in Belgium were published [1]. These recommendations were prepared by a group of Belgian epilepsy experts, based on guidelines for the treatment of epilepsies published by the International League against Epilepsy (ILAE, 2006 [2]), the American Academy of Neurology (AAN, 2004 [3, 4]), the Scottish Intercollegiate Guidelines Network (SIGN, 2003 [5]), and the UK National Institute for Clinical Excellence (NICE, 2004 [6]), and relevant publications of controlled clinical trials with AEDs published after the cut-off dates used in these guidelines. The information from the published guidelines and relevant clinical studies was evaluated and translated into treatment recommendations for the Belgian situation, taking into account the registration status and reimbursement of the compounds and clinical practice in Belgium.

The discussions resulted in recommendations for initial monotherapy and add-on treatment in adults and for initial monotherapy in paediatric patients (<16 years) [1].

In November 2011, the validity of these recommendations was reviewed by the same group of Belgian epilepsy experts that contributed to the preparation of the original paper. Prior to this expert meeting, the scientific literature was searched for international treatment guidelines for epilepsy and relevant controlled clinical trials with AEDs published after preparation of the recommendations in 2008. The criteria used to determine the relevance of the clinical trials were the same as in 2008 [1]. No updates on the ILAE, AAN, SIGN, or NICE guidelines on the treatment of epilepsies have been published since 2008. To the experts’ knowledge, no new guidelines for epilepsy treatment have been issued by any other scientific body either.

In a recently published randomized controlled study by Glauser et al. [7], it was shown that ethosuximide and valproic acid are more effective than lamotrigine in the treatment of childhood absence epilepsy, with ethosuximide being associated with fewer adverse attentional effects. Based on this study the authors are of the opinion that both ethosuximide and valproate should be considered first choice in childhood absence epilepsy. This represents the only change in the 2008 treatment recommendations for monotherapy in paediatric patients.

Since the publication of the 2008 recommendations, rufinamide and stiripentol were registered in Belgium; rufinamide for add-on treatment of patients with Lennox–Gastaut syndrome and stiripentol as adjunctive therapy in patients with Dravet’s syndrome. As the 2008 recommendations only covered initial monotherapy in paediatric patients, these registrations do not imply a change of the recommendations for children.

The present paper focuses on an update of the recommendations for initial monotherapy and for add-on treatment in adult patients.

The following criteria were used to prepare the treatment recommendations in this update:

- The AED is registered and reimbursed in Belgium.
- The AED with the highest level of evidence for efficacy is recommended as first choice. The definitions for the level of evidence were described in detail in the 2008 recommendations [1], and were taken from the ILAE guidelines for monotherapy [2] and the AAN guidelines for add-on treatment [4].
- If the level of evidence for different AEDs is the same or if there is only limited evidence, recommendations are based on personal views and experiences of the authors.

The following definitions are used in the present paper:

- *First choice* First treatment choice
- *Alternative first choice* Compound recommended when certain patient factors (e.g. comorbidity, concomitant medication) or AED-related factors (e.g. pharmacokinetic properties, interaction potential, contraindications, adverse effect profile) preclude the use of the first choice compound.

## Recommendations for treatment

The literature search for papers on monotherapy or add-on treatment with AEDs published since 2008 did not reveal any relevant controlled clinical trials that would lead to a change in the level of evidence for efficacy of the compounds included in Tables 2 and 3.

### Initial monotherapy in adults (≥16 years)

Recommendations for initial monotherapy of seizures in adults are presented in Table 2.

#### Focal seizures with/without secondary generalization

Registered and reimbursed treatment options for monotherapy of focal seizures with or without secondary generalization are carbamazepine, lamotrigine, levetiracetam, pheneturide, phenobarbital, phenytoin, primidone, topiramate, and valproate (Table 1). Since the publication of the recommendations in 2008, no new treatment options have become available for this indication.

**Table 1:** Reimbursed and not reimbursed indications of AEDs (ATC code N03) registered in Belgium

AED	Focal seizures with/without secondary generalization	Primary generalized seizures			Other reimbursed indications	Approved but not reimbursed indications^a^
		Primary generalized tonic–clonic seizures	Juvenile myoclonic epilepsy	Other		
Carbamazepine	Mono- and add-on therapy: adults + children	Mono- and add-on therapy: adults + children				
Eslicarbazepine^b^						Eslicarbazepine is indicated as adjunctive therapy in adults with focal seizures with or without secondary generalisation
Ethosuximide				Absence epilepsy; atonic seizures, myoclonia		
Felbamate					Add-on treatment in patients with Lennox–Gastaut syndrome in adults and children ≥4 years (when not responding to any other relevant AED)	
Gabapentin	Add-on treatment: adults + children^c^					Monotherapy of partial-onset epilepsy with/without secondary generalization in adults and children >12 years
Lacosamide	Add-on treatment: adults + children ≥16 years after failure of therapy with at least 3 AEDs					
Lamotrigine	Monotherapy: adults + children ≥12 years Add-on treatment: adults + children^d^	Monotherapy: adults + children ≥12 years Add-on treatment: adults + children^a^				Add-on treatment of Lennox–Gastaut syndrome in adults and children^e^
Levetiracetam	Monotherapy: adults + children ≥16 years Add-on treatment: adults + children ≥1 month		Add-on treatment: adults and children ≥12 years			Add-on treatment of primary generalized tonic–clonic seizures in adults and children >12 years
Oxcarbazepine	Add-on therapy: adults + children ≥ 6 years					Monotherapy of partial-onset epilepsy with/without secondary generalization in adults and children >6 years
Pheneturide	Mono- and add-on therapy: adults + children ≥2 years					
Phenobarbital^f^	Mono- and add-on therapy: adults + children	Mono- and add-on therapy: adults and children				
Phenytoin	Mono- and add-on therapy: adults + children	Mono- and add-on therapy: adults + children				
Pregabalin	Add-on treatment: adults					
Primidone	Mono- and add-on therapy: adults + children	Mono- and add-on therapy: adults + children	Mono- and add-on therapy: adults and children			
Retigabine	Add-on treatment: adults + children ≥18 years after failure of therapy with at least 3 AEDs					
Rufinamide^g^					Add-on treatment in patients with Lennox–Gastaut syndrome in adults and children ≥4 years (after failure of at least 2 treatments with monotherapy or combinations including valproate or topiramate and/or lamotrigine)	
Stiripentol					Combination treatment with valproate and clobazam of patients with Dravet’s syndrome (severe myoclonic epilepsy of infancy) when seizures are insufficiently controlled by valproate/clobazam	
Tiagabine	Add-on treatment: adults + children ≥12 years					
Topiramate	Monotherapy: adults + children ≥6 years Add-on treatment: adults + children ≥2 years	Monotherapy: adults + children ≥6 years Add-on treatment: adults + children ≥2 years			Add-on treatment in patients with Lennox–Gastaut syndrome in adults and children ≥2 years	
Valproate	Mono- and add-on therapy: adults + children	Mono- and add-on therapy: adults + children	Mono- and add-on therapy: adults + children	Mono- and add-on therapy for absence epilepsy in adults and children	Mono- and add-on therapy in patients with Lennox–Gastaut syndrome, West syndrome	
Vigabatrin	Add-on treatment (last choice): adults + children				Monotherapy of infantile spasms (West syndrome)	
Zonisamide^h^						Zonisamide is indicated as adjunctive therapy in the treatment of adult patients with partial seizures, with or without secondary generalization

Source: FAGG/AFMPS 14-12-2011[Federaal Agentschap voor Geneesmiddelen en Gezondheidsproducten/Agence Fédérale des Médicaments et des Produits de Santé (http://www.fagg-afmps.be)]/RIZIV/INAMI 01-01-2012 [Rijksinstituut voor ziekte- en invaliditeitsverzekering (RIZIV); Institut national d’assurance maladie-invalidité (INAMI); (http://www.inami.fgov.be; http://www.riziv.fgov.be)]

^a^The mentioned indications are not always registered for all brands/products

^b^Eslicarbazepine (Exalief; Zebinix) is not (yet) marketed in Belgium

^c^Some brands specify an age limit of >6 years. Reimbursement criteria do not specify an age limit

^d^Age limit varies among brands

^e^For some brands limited to children >2 years

^f^Magisterial preparation for paediatric use, fully reimbursed

^g^Rufinamide (Inovelon) is not (yet) marketed in Belgium

^h^Zonisamide (Zonegran) is not (yet) marketed in Belgium

*Carbamazepine* and *levetiracetam* are recommended as first choice. Both compounds have level A evidence for efficacy [2, 8]. In 2008 levetiracetam was considered alternative first choice (i.e. compound recommended when certain patient factors or compound-related factors precluded use of first choice carbamazepine) because of the limited clinical experience with this compound in monotherapy. Since 2008 clinical experience with levetiracetam has increased considerably. Therefore, both levetiracetam and carbamazepine now are considered first choice for monotherapy of focal seizures with or without secondary generalization.

*Lamotrigine*, *oxcarbazepine*, *topiramate* and *valproate* are alternative first choices, with level C or B evidence for efficacy in the general adult population. For lamotrigine level A evidence for efficacy was demonstrated in elderly patients [2].

#### Primary generalized seizures

The classification “primary generalized seizures” includes tonic–clonic seizures, absences, myoclonic seizures, clonic seizures, tonic seizures and atonic seizures [11]. The registered (and reimbursed) indications of AEDs do not cover all these subtypes of primarily generalized seizures. Registered and reimbursed AEDs for monotherapy of primary generalized tonic–clonic seizures are carbamazepine, lamotrigine, phenobarbital, phenytoin, primidone, topiramate, and valproate. Primidone and valproate are registered and reimbursed for juvenile myoclonic epilepsy (Table 1).

The authors consider *valproate* to be first choice for the treatment of primary generalized seizures, except in women of childbearing age. *Lamotrigine*, *levetiracetam* and *topiramate* are alternative first choices (Table 2). The level of evidence for efficacy of the AEDs in the treatment of primary generalized seizures is not known. Clinical studies have focused on particular subtypes, such as primary generalized tonic–clonic seizures or JME. The efficacy of lamotrigine is best documented against primarily generalized tonic–clonic seizures, absence seizures, and drop attacks associated with Lennox–Gastaut syndrome [15]. It is less effective than valproate in syndromes associated with myoclonic manifestations or absences [15], and may induce or aggravate myoclonic seizures. The efficacy of levetiracetam is best documented against primarily generalized tonic–clonic seizures, and myoclonic seizures [15]; information on its efficacy against tonic and atonic seizures is lacking. The efficacy of topiramate is best documented against primarily generalized tonic–clonic seizures and drop attacks associated with Lennox–Gastaut syndrome. There is no information available on the efficacy of topiramate against absence seizures.

**Table 2:** Initial monotherapy of seizures in adults (≥16 years)

	First choice	Level of evidence for efficacy	Alternative first choice	Level of evidence for efficacy	Remarks
Focal seizures with/without secondary generalization	Carbamazepine Levetiracetam	A	Valproate	B	*Levetiracetam* has a better pharmacokinetic and safety profile than *carbamazepine*, with no potential for drug interactions For *lamotrigine* the overall level of evidence for efficacy is C. Level A evidence for efficacy was obtained in elderly patients
			Lamotrigine Oxcarbazepine Topiramate	C	
Primary generalized seizures	Valproate	–	Lamotrigine Levetiracetam Topiramate	–	The efficacy of *lamotrigine* is best documented against primarily generalized tonic–clonic seizures, absence seizures, and drop attacks associated with Lennox–Gastaut syndrome [15] Lamotrigine may induce or aggravate myoclonic seizures The efficacy of *levetiracetam* is best documented against primarily generalized tonic–clonic seizures, and myoclonic seizures. Efficacy against tonic and atonic seizures has not been documented [15] The efficacy of *topiramate* is best documented against primarily generalized tonic–clonic seizures and drop attacks associated with Lennox–Gastaut syndrome. Efficacy against absence seizures has not been documented [15]

*First choice* first treatment choice, *Alternative first choice* compound recommended when certain patient factors (e.g. comorbidity, concomitant medication) or compound-related factors (e.g. pharmacokinetic properties, interaction potential, adverse effect profile) preclude the use of the first choice compound, *Level of evidence for efficacy* the criteria used to establish the level of evidence for efficacy are taken from Glauser et al. [7]

*Carbamazepine* may be a valuable alternative first choice for the treatment of primary generalized tonic–clonic seizures, but is not effective for other types of primary generalized seizures, and may induce or aggravate myoclonic seizures and absences.

#### Type of seizures not (yet) established

If starting the AED treatment is required prior to establishing seizure type, a broad spectrum AED should be used, such as *valproate, lamotrigine, levetiracetam* and *topiramate*, taking into account the relative benefits and risks of each of these compounds.

### Add-on treatment of seizures in adult patients (≥16 years)

In the consensus proposal of the ILAE Commission on Therapeutic Strategies, published in 2010, drug-resistant epilepsy is defined as “failure of adequate trials of two tolerated and appropriately chosen and used AED schedules (whether as monotherapies or in combination) to achieve sustained seizure freedom” [9].

Combination/add-on therapy can be beneficial in patients who did not respond to monotherapy. Compounds recommended for add-on treatment are presented in Table 3. *Carbamazepine*, *gabapentin*, *lacosamide*, *lamotrigine*, *levetiracetam*, *oxcarbazepine*, *pregabalin*, *retigabine*, *tiagabine*, *topiramate*, and *valproate* are all considered first choice for add-on treatment of focal seizures with or without secondary generalization. For all newer AEDs the level of evidence for efficacy is A. No add-on studies have been performed with the older AEDs, but the efficacy of these compounds is considered to be established during long-term clinical experience. Lacosamide and retigabine are reimbursed for patients in whom treatment with at least 3 other AEDs has failed. The other registered and reimbursed AEDs (pheneturide, phenytoin, phenobarbital, primidone, and vigabatrin) are not recommended, because of their unfavourable pharmacokinetic and/or safety profile. *Eslicarbazepine* 1 and *zonisamide* 2 are registered for add-on treatment of focal seizures, but are not yet marketed/reimbursed in Belgium.

**Table 3:** Add-on treatment of seizures in adults (≥16 years)

	Recommended AEDs	Remarks
Focal seizures with/without secondary generalization	Carbamazepine	All AEDs are efficacious as add-on treatment of epilepsy in adults^a^, and are considered first choice. The AEDs are listed in alphabetical order *Carbamazepine* has been used in clinical practice for over 30 years, but has a high potential for pharmacokinetic interactions *Gabapentin, levetiracetam* and *pregabalin* have the most favourable pharmacokinetic and safety profile, and no potential for drug interactions *Vigabatrin* is also registered and reimbursed for add-on treatment of partial-onset epilepsy, but should only be used when all other compounds are ineffective, because it has a very unfavourable safety profile (concentric visual field defects) *Lacosamide* and *retigabine* are reimbursed for patients in whom treatment with at least 3 other AEDs has failed
	Gabapentin	
	Lacosamide	
	Lamotrigine	
	Levetiracetam	
	Oxcarbazepine	
	Pregabalin	
	Retigabine	
	Tiagabine	
	Topiramate	
	Valproate	
Primary generalized tonic–clonic seizures	Carbamazepine	All AEDs are efficacious as add-on treatment of primary generalized tonic–clonic seizures in adults^a,^ and are considered first choice. The AEDs are listed in alphabetical order
	Lamotrigine	
	Levetiracetam	
	Topiramate	
	Valproate	

^a^For all newer AEDs the level of evidence for efficacy is A (the criteria used to establish the level of evidence for efficacy are taken from French et al. [3, 4]. No add-on studies have been performed with the older AEDs; efficacy of these compounds is considered to be established during long-term clinical experience

*Carbamazepine*, *lamotrigine*, *levetiracetam*, *topiramate* and *valproate* are recommended as first choice for add-on treatment of primary generalized tonic–clonic seizures. For lamotrigine, levetiracetam and topiramate the level of evidence for efficacy is A. Efficacy of carbamazepine and valproate is considered to be established during long-term clinical experience. Phenytoin and primidone are not recommended as first choice for this indication, because of their unfavourable pharmacokinetic and/or safety profile.

The large number of possible combinations of two or more AEDs has led to an increased interest in combination strategies. The goals of “rational polytherapy” are to maximize seizure control and minimize adverse effects. Ideal combinations are those which display pharmacodynamic synergism, which ideally may lead to improved efficacy without a proportional increase in toxicity [12, 13].

Theoretically it may be assumed that combining AEDs with different mechanisms of action will provide a better potential for additive or even synergistic efficacy and/or a more favourable tolerability profile, and may also be more likely to be effective against a broad range of seizure types in patients with refractory epilepsy compared to combining AEDs with the same mechanism of action [12–14]. There are some indications from the literature on animal models of seizures and epilepsy to confirm this view [12–14], but convincing evidence from non-clinical or clinical studies is lacking.

It should be noted that the mechanism-of-action-based “rational polytherapy” approach is seriously hampered by the lack of knowledge on the processes underlying seizure generation and propagation, and the lack of knowledge on the exact mechanism(s) of action of most AEDs, with many AEDs having multiple pharmacodynamic effects [13].

There is more scientific evidence in favour of pharmacodynamic interaction with respect to adverse effects. When adverse effects of co-administered drugs are similar, combining these drugs may lead to a “threshold” of tolerability being exceeded for that particular side effect. Use of combinations of drugs that block voltage-dependent sodium channels (carbamazepine, lacosamide, lamotrigine, oxcarbazepine) is more likely to produce neurotoxic side effects, such as dizziness, diplopia and ataxia [12, 14]. Since robust evidence to support rational polytherapy is still very limited, the choice of drug combinations in clinical practice will have to be tailored on a case-by-case basis [13].

## Specific considerations

### Pharmacokinetic properties and pharmacokinetic interaction profile

Most patients with epilepsy are treated for several years and many need life-long treatment with AEDs. Several patients may be treated with more than one AED, and the likelihood of concomitant treatment with drugs for other diseases (both related and not related to epilepsy) at any point in their lives is high. The absence of a potential for drug interactions is therefore an important positive feature of any AED, which will highly increase its ease of use.

Information on the pharmacokinetic profile of all AEDs included in Tables 2 and 3 is presented in Table 4. The pharmacokinetic profile ratings are taken from the handbook on epilepsy and epilepsy treatment by Panayiotopoulos [16]. The pharmacokinetic profile rating is based on the rating system described by Patsalos [17]. The pharmacokinetic characteristics included in this rating system are: oral absorption (speed of absorption, bioavailability, affected or not by food), kinetics (linearity, saturability), extent of plasma protein binding, extent of renal elimination, metabolism (hepatic, inducible, autoinducible, active metabolites), pharmacokinetic drug interactions (affected by other AEDs, affects other AEDs, affected by other drugs, affects other drugs), and dosing frequency. All parameters are scored on a 3-point rating scale, with 3 being the most favourable score. The score presented in Table 4 is expressed as a percentage of the maximum possible score. Lacosamide and levetiracetam have the highest score (96 %), followed by gabapentin and pregabalin (both 89 %). Carbamazepine and valproate have the lowest scores (50 and 52 %, respectively).

**Table 4:** Pharmacokinetic profile rating and pharmaceutical formulations of the AEDs recommended in Tables 2 and 3

AED	Pharmacokinetic profile rating of AEDs^a^	Pharmaceutical formulations
Carbamazepine	50	Tablets Oral solution/syrup
Gabapentin	89	Tablets Capsules
Lacosamide	96	Tablets I.V. formulation^b^
Lamotrigine	73	Orodispersible tablet
Levetiracetam	96	Tablet Oral solution I.V. formulation
Oxcarbazepine	77	Tablets
Pregabalin	89	Capsules
Retigabine	NA	Tablets
Tiagabine	67	Tablets
Topiramate	79	Tablets Capsules
Valproate	52	Capsules Controlled-release capsules Controlled-release tablets Enteric-coated capsules Oral solution/syrup I.V. formulation

*NA* no information available

^a^Data taken from Panayiotopoulos [16]; 3-point rating system based on the following parameters: oral absorption, kinetics, plasma protein binding, elimination metabolism, drug interactions and dosing frequency (see Patsalos [17])

^b^Not reimbursed

The use of an AED with a high potential for pharmacokinetic interactions may alter the plasma concentrations of the other AEDs in the combination, thereby affecting their efficacy or increasing the risk of side effects.

### Tolerability

Treatment with AEDs may be associated with adverse effects. For a complete overview of all adverse effects, the reader is referred to the summaries of product characteristics (SmPCs) of the individual AEDs. Handbooks, review articles and other publications may vary considerably with respect to their opinions on the most important or clinically most relevant adverse effects. Table 5 presents the most common adverse effects (occurring in more than 10 % of the patients, as listed in the SmPCs of the AEDs) and “other important adverse events” (mentioned in at least 3 of 5 recently published reviews [10, 18–21]) of the AEDs listed in Tables 2 and 3.

**Table 5:** Most important adverse effects of the AEDs recommended in Tables 2 and 3

AED	Most common adverse effects (occurring in >10 % of the patients)^a^	Other important adverse events^b^
Carbamazepine	Central nervous system: dizziness, ataxia, sleepiness Gastrointestinal: nausea, vomiting Skin: allergic dermatitis, urticaria (may become serious) Other: leukopenia, tiredness, increased gamma-GT levels (usually not clinically relevant)	Diplopia Weight gain Hyponatraemia (aplastic) anaemia Serious dermatologic reactions (Stevens–Johnson syndrome)
Gabapentin	Central nervous system: dizziness, ataxia, sleepiness Other: viral infection, tiredness, fever	Weight gain
Lacosamide	Central nervous system: dizziness Gastrointestinal: nausea Other: headache, diplopia	Dose-related increase in PR-interval (atrioventricular block)
Lamotrigine	Central nervous system: dizziness, ataxia, somnolence Gastrointestinal: nausea, vomiting Skin: rash Other: headache, diplopia, blurred vision	Insomnia Serious dermatologic reactions (Stevens–Johnson syndrome) Hypersensitivity syndrome
Levetiracetam	Central nervous system: somnolence Other: asthenia (tiredness)	Dizziness Aggressive behaviour (irritability, hostility)
Oxcarbazepine	Central nervous system: dizziness, sleepiness Gastrointestinal: nausea, vomiting Other: headache, tiredness, diplopia	Ataxia Rash Serious dermatologic reactions (Stevens–Johnson syndrome) Hyponatraemia
Pregabalin	Central nervous system: dizziness, sleepiness	Weight gain
Retigabine	Central nervous system: dizziness, somnolence Other: fatigue	Urinary retention QT interval prolongation Confusional state, psychotic symptoms and hallucinations
Tiagabine	Central nervous system: dizziness, somnolence, depressed mood, nervousness, concentration disturbances, tremor Other: tiredness	–
Topiramate	Central nervous system: dizziness, sleepiness, depression, paraesthesia Gastrointestinal: diarrhoea, nausea Other: tiredness, weight loss, nasopharyngitis	Speech disorders Metabolic acidosis Kidney stones Oligohidrosis Glaucoma
Valproate	Central nervous system: tremor Gastrointestinal: nausea, vomiting, indigestion Other: weight gain, hair loss	Thrombocytopenia Hepatotoxicity Acute pancreatitis Hyperammonaemia

For a complete overview of adverse events the reader is referred to the SmPCs of the individual products

^a^Taken from the SmPCs of the individual AEDs. As for valproate no incidence of adverse events is given in the SmPC, the listed adverse effects are those considered by the experts to be most common

^b^Taken from 5 recently published reviews [10, 18–21]. The listed “other important adverse events” are those mentioned in at least 3 of these 5 published sources. For lacosamide and retigabine the “other important adverse events” are taken from the SmPC

The adverse effect profile of an AED is a relevant factor for selecting the optimal compound for an individual patient. It is for instance not advisable to treat elderly patients with AEDs with a considerable sedative effect. Compounds known to induce depression or psychosis should be avoided in patients with a history of psychiatric conditions.

The adverse effect profile of AEDs is also relevant when selecting AEDs for combination therapy (see “Add-on treatment of seizures in adult patients”).

### Comorbidity

Epilepsy is often associated with other CNS-related conditions, such as anxiety, depression, migraine, sexual disorders and cognitive problems [22]. In addition, patients with epilepsy may also suffer from health problems not related to their epilepsy. The presence of concomitant diseases should be taken into account when selecting an AED, since they may form an absolute or relative contraindication to the use of certain AEDs (for more information the reader is referred to the SmPCs of the individual AEDs).

### Elderly

Epilepsy is a common neurologic disorder in the elderly. The most common causes of new-onset seizures in this age group are cerebrovascular disease, neurodegenerative disorders and brain tumours, leading to focal seizures with or without generalization. *Carbamazepine*, *lamotrigine* and *gabapentin* have level A evidence for efficacy for initial monotherapy of this type of seizures in the elderly [2, 23]. It should be noted that gabapentin is not reimbursed for monotherapy. Results of open-label studies indicate that *levetiracetam* [24–27] *topiramate* [28–30] and *oxcarbazepine* [31–33] are also efficacious and safe in elderly patients.

Choosing the optimal AED for an elderly patient is complicated, because of the frequent presence of comorbid diseases (such as osteoporosis, cognitive deterioration, parkinsonism, and renal and/or hepatic insufficiency) and use of (chronic) concomitant medication. Altered pharmacokinetics and a higher susceptibility to the adverse effects of AEDs, particularly to those related to the nervous system, should be taken into account. Compounds with a high potential for drug interactions, for instance due to induction of metabolic enzymes (such as carbamazepine), and AEDs having a high probability of adverse effects on cognition (such as topiramate) should be avoided.

AED treatment in elderly patients should be done with caution. Dose escalation should be done very carefully, and maintenance doses will probably be lower than usual in many cases.

### Pregnancy

The risks of AED use during pregnancy are of major concern. In 2009, the American Academy of Neurology (AAN) published 3 reports on management issues for women with epilepsy, based on an evaluation of relevant articles published between 1985 and 2008 [34–36]. These reports addressed the following topics: obstetric complications and change in seizure frequency [34], teratogenesis and perinatal outcome [35], preconceptional folic acid and prenatal vitamin K use, the clinical implications of placental and breast-milk transfer of AEDs, and the effects of pregnancy on AED plasma levels (including the necessity of monitoring AED plasma concentrations during pregnancy) [36].

Additional information has become available since the issue of these guidelines (e.g. [37, 38].), but there is still a considerable lack of knowledge about the effects of AEDs on the foetus, with the relative risks of the individual compounds remaining poorly understood. Information from population-based studies and from data collected by various pregnancy registries worldwide will have to fill the knowledge gaps.

Discontinuation of AED treatment prior to or during pregnancy is usually not an option. It is recommended to review AED treatment prior to conception, and, if possible, use monotherapy with the most effective AED at the lowest effective dose. To avoid high plasma concentrations the use of slow-release preparations or a multiplication of the frequency of oral intake may be considered for some AEDs.

Several publications indicate an increased risk of major foetal malformations [35, 38–41], and an increased risk of delayed early cognitive development [35, 41–43] associated with the use of valproate during pregnancy.

Therefore, use of valproate (particularly at higher dose levels) and AED polytherapy (particularly combinations including valproate) during the first trimester of pregnancy should be avoided, to reduce the risk of major congenital malformations. If possible, valproate and AED polytherapy should be avoided throughout pregnancy to prevent reduced cognitive outcomes.

Pregnancy probably causes a decrease in the plasma concentrations of lamotrigine, phenytoin, and to a lesser extent of carbamazepine. Plasma concentrations of levetiracetam and of the active metabolite of oxcarbazepine (10-monohydroxy derivative) may also be decreased [36]. Monitoring plasma concentrations of lamotrigine, carbamazepine, levetiracetam and oxcarbazepine (as its 10-monohydroxy derivative) during pregnancy and after child birth (for monitoring the risk of overdose) should be considered. Comparison of plasma concentrations before and during pregnancy will reveal pregnancy-induced alterations in pharmacokinetics of the compound(s) in the individual patient, and may provide a basis for dose adjustments [41].

Since part of the teratogenicity of AEDs may be related to an AED-induced decrease in folic acid levels (due to decreased absorption and increased excretion), supplementation with folic acid (0.4–5 mg/day; in Belgium the commonly prescribed dose is 4 mg/day) prior to conception and during the first trimester of pregnancy is recommended [36].

### Generic substitution

To reduce the cost of health care the use of generics is strongly advocated by health insurance companies and governmental institutions. Generic substitution of AEDs is, however, not without risks.

Approval of generic products is based on bioequivalence with the original (brand) product. Two products are considered to be bioequivalent if the 90 % confidence interval for the ratio of test and reference product for AUC_0–*t*_ and *C*_max_ falls within the acceptance interval of 80.00–125.00 % [44]. Though all generic AEDs are bioequivalent with the original (brand) product, there may be large differences in plasma concentrations between two generic products, if the bioavailability of the two products is at the boundaries of the acceptance interval [45]. Moreover, the design of the studies used to investigate bioequivalence (mainly single-dose studies in healthy volunteers under highly standardized conditions), do not guarantee that the products concerned will also be bioequivalent in an individual patient during chronic use [46]. The situation is even more complicated for compounds with non-linear kinetics (such as phenytoin), or when there is no clear correlation between plasma concentration and therapeutic effect.

When considering generic substitution of an AED, the following should be taken into account. Possible differences in bioavailability between brands may lead to loss of seizure control (recurrence of seizures) [47, 48], with major therapeutic and social consequences for the patient, such as career restrictions or even loss of employment or loss of driving license [48, 49]. Differences in bioavailability, and particularly an increase in peak plasma levels, may lead to an increase in the frequency and severity of adverse effects [47, 49].

Differences in appearance of the package or in colour, shape or taste of the product may confuse the patient, thereby leading to lower treatment compliance, with the possibility of break-through seizures [48, 49].

The consequences of generic substitution, such as re-appearance of seizures and/or an increase in frequency or severity of adverse effects may lead to additional health care costs, which may largely surpass the initial savings earned with the generic substitution [48, 49].

The Belgian Center for Pharmacotherapeutic Information (BCFI-CBIP) considers AEDs to be compounds with a narrow therapeutic margin [50]. In the information provided on the BCFI-CBIP website all AEDs are categorized as “No Switch”, indicating that switching between brands and generics is not recommended [51].

Therefore, when a patient is successfully treated with a particular brand of an AED, it is advised to continue treatment with that same compound. When choosing between the brand or one of the generic versions of an AED the likelihood of continuous supply of the compound from the same manufacturer should be taken into account [15, 46]. Prescription of an AED based solely on the active substance by International Non-proprietary Name (INN), without any indication of the brand or manufacturer, should be avoided.

## Footnotes

1 Exalief; Zebinix.

2 Zonegran.

## References

[1] Boon P, Engelborghs S, Hauman H, Jansen A, Lagae L, Legros B, Ossemann M, Sadzot B, Urbain E, van Rijckevorsel K (2008). Recommendations for the treatment of epilepsies in general practice in Belgium. Acta Neurol Belg.

[2] Glauser T, Ben-Menachem E, Bourgeois B, Cnaan A, Chadwick D, Guerreiro C (2006). ILAE treatment guidelines: evidence-based analysis of antiepileptic drug efficacy and effectiveness as initial monotherapy for epileptic seizures and syndromes. Epilepsia.

[3] French JA, Kanner AM, Bautista J, Abou-Khalil B, Browne T, Harden CL (2004). Efficacy and tolerability of the new antiepileptic drugs I: treatment of new onset epilepsy. Report of the Therapeutics and Technology Assessment Subcommittee and Quality Standards Subcommittee of the American Academy of Neurology and the American Epilepsy Society. Neurology.

[4] French JA, Kanner AM, Bautista J, Abou-Khalil B, Browne T, Harden CL (2004). Efficacy and tolerability of the new antiepileptic drugs II: treatment of refractory epilepsy: report of the Therapeutics and Technology Assessment Subcommittee and Quality Standards Subcommittee of the American Academy of Neurology and the American Epilepsy Society. Neurology.

[5] Scottish Intercollegiate Guidelines Network. Diagnosis and management of epilepsy in adults. A national clinical guideline. April 2003. ISBN:1 899893 58 X

[6] NHS. National Institute for Clinical Excellence. The epilepsies. The diagnosis and management of the epilepsies in adults and children in primary and secondary care. Clinical Guideline 20, October 2004. ISBN:1-84257-808-1

[7] Glauser TA, Cnaan A, Shinnar S, Hirtz DG, Dlugos D, Masur D, for the Childhood Absence Epilepsy Study Group (2010). Ethosuximide, valproic acid, and lamotrigine in childhood absence epilepsy. N Engl J Med.

[8] Brodie MJ, Perucca E, Ryvlin P, Ben-Menachem E, Meencke HJ, for the Levetiracetam Monotherapy Study Group (2007). Comparison of levetiracetam and controlled-release carbamazepine in newly diagnosed epilepsy. Neurology.

[9] Kwan P, Arzimanoglou A, Berg AT, Brodie MJ, Hauser WA, Mathern G (2010). Definition of drug resistant epilepsy: consensus proposal by the ad hoc Task Force of the ILAE Commission on Therapeutic Strategies. Epilepsia.

[10] Toledano R, Gil-Nagel A (2008). Adverse effects of antiepileptic drugs. Semin Neurol.

[11] Berg AT, Berkovic SF, Brodie MJ, Buchhalter J, Cross H, van Emde Boas W (2010). Revised terminology and concepts for organization of seizures and epilepsies: report of the ILAE Commission on Classification and Terminology, 2005–2009. Epilepsia.

[12] Brodie MJ, Sills GJ (2011). Combining antiepileptic drugs—rational polytherapy?. Seizure.

[13] Lee JW, Dworetzky B (2010). Rational polytherapy with antiepileptic drugs. Pharmaceuticals.

[14] French JA, Faught E (2009). Rational polytherapy. Epilepsia.

[15] Perucca E, Tomson T (2011). The pharmacological treatment of epilepsy in adults. Lancet Neurol.

[16] Panayiotopoulos CP (2010) A clinical guide to epileptic syndromes and their treatment. Revised second edition. Based on the ILAE classifications and practice parameter guidelines. Springer Healthcare, p 178

[17] Patsalos PN (2005). Properties of antiepileptic drugs in the treatment of idiopathic generalized epilepsies. Epilepsia.

[18] Tatum WO (2010). Antiepileptic drugs: adverse effects and drug interactions. Neurology.

[19] Cramer JA, Mintzer S, Wheless J, Mattson RH (2010). Adverse effects of antiepileptic drugs: a brief overview of important issues. Expert Rev Neurother.

[20] French JA, Gazzola DM (2011). New generation antiepileptic drugs: what do they offer in terms of improved tolerability and safety?. Ther Adv Drug Saf.

[21] Panayiotopoulos CP (2010) A clinical guide to epileptic syndromes and their treatment. Revised second edition. Based on the ILAE classifications and practice parameter guidelines. Springer Healthcare, pp 569–607

[22] Elger CE, Schmidt D (2008). Modern management of epilepsy: a practical approach. Epilepsy Behav.

[23] Rowan AJ, Ramsay RE, Collins JF, Pryor F, Boardman KD, Uthman BM (2005). New onset geriatric epilepsy. A randomized study of gabapentin, lamotrigine and carbamazepine. Neurology.

[24] Belcastro V, Costa C, Galletti F, Autuoria A, Pierguidi L, Pisani F (2008). Levetiracetam in newly diagnosed late-onset post-stroke seizures: a prospective observational study. Epilepsy Res.

[25] Kutlu G, Gomceli YB, Unal Y, Inan LE (2008). Levetiracetam monotherapy for late poststroke seizures in the elderly. Epilepsy Behav.

[26] Lippa CF, Rosso A, Hepler M, Jenssen S, Pillai J, Irwin D (2010). Levetiracetam: a practical option for seizure management in elderly patients with cognitive impairment. Am J Alzheimers Dis Other Dement.

[27] García-Escrivá A, López-Hernández N (2007). Uso del levetiracetam en monoterapia en crisis postictus de la población anciana (The use of levetiracetam in monotherapy in post-stroke seizures in the elderly population). Rev Neurol.

[28] Stefan H, Hubbertz L, Peglau I, Berrouschot J, Kasper B, Schreiner A, Krimmer J, Schauble B, on behalf of the TOP-GER-13 investigators (2008). Epilepsy outcomes in elderly treated with topiramate. Acta Neurol Scand.

[29] Grošelj J, Guerrini R, Van Oene J, Lahaye M, Schreiner A, Schwalen S, the TOP-INT-51 Investigators’ Group (2005). Experience with topiramate monotherapy in elderly patients with recent-onset epilepsy. Acta Neurol Scand.

[30] Runge U, Schaeuble B, Rettig K, Schreiner A (2007). Topiramat—Initiale Monotherapie bei älteren Patienten mit Epilepsie. (Topiramate—first line monotherapy in elderly patients with epilepsy). Akt Neurol.

[31] Kutluay E, McCague K, D’Souza J, Beydoun A (2003). Safety and tolerability of oxcarbazepine in elderly patients with epilepsy. Epilepsy Behav.

[32] Dogan EA, Usta BE, Bilgen R, Senol Y, Aktekin B (2008). Efficacy, tolerability, and side effects of oxcarbazepine monotherapy: a prospective study in adult and elderly patients with newly diagnosed partial epilepsy. Epilepsy Behav.

[33] Freidel M, Krause E, Kuhn K, Peper R, Vogel H (2007). Oxcarbazepin in der Epilepsietherapie: Multizentrische Anwendungsbeobachtung mit 1385 Epilepsie-Patienten zur Neueinstellung oder Umstellung auf Oxcarbazepin. (Oxcarbazepine in the treatment of epilepsy: an open-labelled, prospective, post-marketing surveillance study with oxcarbazepine in the treatment of 1385 patients with epilepsy). Fortschr Neurol Psychiat.

[34] Harden CL, Hopp J, Ting TY, Pennell PB, French JA, Hauser WA (2009). Management issues for women with epilepsy—focus on pregnancy (an evidence-based review): I. Obstetrical complications and change in seizure frequency. Report of the Quality Standards Subcommittee and Therapeutics and Technology Assessment Subcommittee of the American Academy of Neurology and the American Epilepsy Society. Epilepsia.

[35] Harden CL, Meador KJ, Pennell PB, Hauser WA, Gronseth GS, French JA (2009). Management issues for women with epilepsy—focus on pregnancy (an evidence-based review): II. Teratogenesis and perinatal outcomes. Report of the Quality Standards Subcommittee and Therapeutics and Technology Assessment Subcommittee of the American Academy of Neurology and the American Epilepsy Society. Epilepsia.

[36] Harden CL, Pennell PB, Koppel B, Hovinga CA, Gidal B, Meador KJ (2009). Management issues for women with epilepsy—focus on pregnancy (an evidence-based review): III. Vitamin K, folic acid, blood levels, and breast feeding. Report of the Quality Standards Subcommittee and Therapeutics and Technology Assessment Subcommittee of the American Academy of Neurology and the American Epilepsy Society. Epilepsia.

[37] Mølgaard-Nielsen D, Hviid A (2011). Newer-generation antiepileptic drugs and the risk of major birth defects. JAMA.

[38] Tomson T, Battino D, Bonizzoni E, Craig J, Lindhout D, Sabers A, Perucca E, Vajda F, EURAP study group (2011). Dose-dependent risk of malformations with antiepileptic drugs: an analysis of data from the EURAP epilepsy and pregnancy registry. Lancet Neurol.

[39] Hernandez-Diaz S, Smith CR, Shen A, Mittendorf R, Holmes LB (2011). Comparative safety of anticonvulsants during pregnancy: seizures or major malformations. Pharmacoepidemiol Drug Saf.

[40] Vajda FJ, Horgan D, Hollingworth S, Graham J, Hitchcock AA, Roten A, et al (2011) The prescribing of antiepileptic drugs for pregnant Australian women. Aust NZ J Obstet Gynaecol. doi:10.1111/j.1479-828X.2011.01359.x 10.1111/j.1479-828X.2011.01359.x 21910695

[41] Tomson T, Battino D (2011). Antiepileptic treatment in pregnant women: morphological and behavioural effects. Handb Exp Pharmacol.

[42] Bromley RL, Mawer G, Love J, Kelly J, Purdy L, McEwan L (2010). Early cognitive development in children born to women with epilepsy. A prospective report. Epilepsia.

[43] Shallcross R, Bromley RL, Irwin B, Bonnett LJ, Morrow J, Baker GA, On behalf of the Liverpool Manchester Neurodevelopment Group and the UK Epilepsy and Pregnancy Register (2011). Child development following in utero exposure. Levetiracetam vs sodium valproate. Neurology.

[44] Committee for medicinal Products for Human Use (CHMP). Guideline on the investigation of bioequivalence. CPMP/EWP/QWP/1401/98 Rev. 1/Corr. (20 January 2010)

[45] Krauss GL, Caffo B, Chang YT, Hendrix CW, Chuang K (2011). Assessing bioequivalence of generic antiepilepsy drugs. Ann Neurol.

[46] Trinka E, Krämer G, Graf M (2011). Requirements for generic antiepileptic medicines: a clinical perspective. J Neurol.

[47] Chaluvadi S, Chiang S, Tran L, Goldsmith CE, Friedman DE (2011). Clinical experience with generic levetiracetam in people with epilepsy. Epilepsia.

[48] Sander JW, Ryvlin P, Stefan H, Booth DR, Bauer J (2010). Generic substitution of antiepileptic drugs. Expert Rev Neurother.

[49] van Paesschen W, Hauman H, Lagae L (2009). The use of generic medication in epilepsy: a review of potential issues and challenges. Eur J Paediatr Neurol.

[50] Belgisch Centrum voor farmacotherapeutische Informatie (Belgian Center for Pharmacotherapeutic Information). Gecommentarieerd Geneesmiddelen Repertorium (Annotated repertory of medicinal products). http://www.bcfi.be. Overschakelen van de ene specialiteit naar de andere (Switching from one brand to another) (February 2006)

[51] Belgisch Centrum voor Farmacotherapeutische Informatie (Belgian Center for Pharmacotherapeutic Information). Gecommentarieerd Geneesmiddelen Repertorium (Annotated repertory of medicinal products). http://www.bcfi.be. July 2011

